# Supplementary material for: Development of a computer-aided design software for the quantitative evaluation of aesthetic damage
Source: PLoS One. 2019 Dec 18;14(12):e0226322. doi: 10.1371/journal.pone.0226322 (PMC6919621; doi:10.1371/journal.pone.0226322)
Supplement: S1 Code site — 1. (ZIP) [file pone.0226322.s001.zip › [EESC_jr][Projeto]Relatorio_07_21/reports/templates_zip/gentelella-master/vendors/Flot/examples/canvas/index.html]

Flot Examples: Canvas text


## Canvas text

This example uses the same dataset (raw oil price in US $/barrel of crude oil vs. the exchange rate from US $ to €) as the multiple-axes example, but uses the canvas plugin to render axis tick labels using canvas text.

Enable canvas text

Copyright © 2007 - 2014 IOLA and Ole Laursen
